# Dietary *Pediococcus acidilactici* improves the intestinal functions by regulating the expression of intestinal inflammatory genes and intestinal microbiota in aged laying hens at 80–91 weeks of age

**DOI:** 10.3389/fmicb.2025.1530319

**Published:** 2025-02-18

**Authors:** Airong Dong, Xuemei Ding, Jianping Wang, Qiufeng Zeng, Shiping Bai, Yan Liu, Yue Xuan, Shanshan Li, Yadong Mu, Huanwei Peng, Keying Zhang

**Affiliations:** Key Laboratory of Animal Disease-Resistance Nutrition, Key Laboratory of Sichuan Province, Ministry of Agriculture and Rural Affairs, Animal Nutrition Institute, Sichuan Agricultural University, Chengdu, China

**Keywords:** *Pediococcus acidilactici*, egg production, gut microbiota, aged laying hens, metabolome

## Abstract

**Introduction:**

*Pediococcus acidilactici* CNCM I-4622 (PA) is a homofermentative Gram-positive coccus that produces lactic acid as a major metabolic by-product. However, the potential of PA to improve intestinal function and, as a result, improve production performance and quality in aged laying hens remains unclear. This study aimed to investigate the effects of PA on egg production, egg quality, intestinal health, and cecal microbiota in aged laying hens.

**Methods:**

A total of 180 Lohmann pink laying hens, aged 80 weeks, were randomly assigned to five groups and fed either a basal diet (PA0) or basal diets supplemented with PA at concentrations of 50, 100, 150, and 200 mg/kg (PA50, PA100, PA150, and PA200).

**Results:**

Compared to the PA0 group, PA did not have a significant effect on the production performance of laying hens (*p* > 0.05). However, the content of diamine oxidase and the expression level of *IL-8* mRNA in the PA50, P100, P150, and P200 groups were significantly reduced (*p* < 0.05). Additionally, the ileal villus height was significantly increased (*p* < 0.05). The cecal chowder pH and ileal crypt depth were also significantly lower (*p* < 0.05), while lipase activity in the ileal mucosa of the PA50 group was significantly increased compared to the PA0 group (*p* < 0.05). Furthermore, the expression of *INF-γ* and *TNF-α* mRNA in the jejunal mucosa was significantly down-regulated (*p* < 0.05), whereas the expression of *Claudin* mRNA was significantly up-regulated (*p* < 0.05). Notably, the relative abundance of *Bacteroidota*, *Fusobacteriota*, and *Fusobacterium* in the PA50 group was significantly higher than that in the PA0 group (*p* < 0.05).

**Discussion:**

Additionally, cecal metabolomic analysis indicated that following the addition of PA, the pathways enriched with differential metabolites were primarily related to arginine and proline metabolism. Therefore, PA has the potential to improve intestinal morphology and flora, mitigate intestinal inflammatory factors, and strengthen intestinal barrier function. These benefits are attributed to the modulation of arginine and proline metabolic pathways, with optimal effects observed at an addition of 50 mg/kg.

## Introduction

Due to improved production capabilities among domestic fowl, the average lifespan of commercial laying hens has increased from 72 to 80 weeks (Liu et al., 2022). However, between 80 and 100 weeks of age, hens experience a decline in both egg production and quality. As a result, they are gradually removed from the flock and referred to as ‘spent hens’ and are classified as aged laying hens (Fan and Wu, 2022). The primary challenge faced by these aged laying hens is the decline in intestinal function (Gan et al., 2020; Abdollahi et al., 2021; Feng et al., 2021).

In recent years, there has been a significant surge of interest in extending the production period of hens. Intestinal health is closely associated with the overall well-being, defense mechanisms, and nutritional status of the host as they age (An et al., 2018). Maintaining gastrointestinal tract health is critically important in contemporary poultry farming practices aimed at enhancing production, especially in laying hens (Fu et al., 2022). The gastrointestinal is the primary pathway for nutrient absorption and the initiation of immune responses. However, this pathway is vulnerable to disruption from a variety of stressors, including environmental fluctuations, pathogens, and changes in feed. Furthermore, the gastrointestinal tract is essential for immune response, microbial defense, and hormonal regulation (Fu et al., 2022). Animal guts are host to trillions of microbes that perform vital functions, including the harvesting, storage, and expenditure of energy derived from dietary sources for the host, effectively acting as an additional organ (Becattini et al., 2016). Disruption of the balance among these microorganisms can adversely affect the health and productivity of hens. Consequently, significant efforts are being made to enhance the production performance of aged laying hens through nutritional interventions. These interventions include supplementing exogenous enzymes, prebiotics, probiotics, and minerals (Kim et al., 2012; Abdelqader et al., 2013b; Ghasemian and Jahanian, 2015). In recent years, interest in probiotics has surged, supported by increasing evidence of their efficacy as a promising approach to enhancing digestive health (Lee et al., 2018; Ji et al., 2019; Cai et al., 2020). Among these probiotics, PA has garnered considerable attention due to its potent physiological activity and resilience. PA represents a category of lactic acid bacteria notable for certain key traits, including its resistance to acidic environments, lack of porphyrin synthesis, and a strictly fermentative (homofermentative) metabolism that is facultatively anaerobic, primarily producing lactic acid as the main metabolic byproduct (Li et al., 2021). Furthermore, PA is recognized as a consistent producer of bacteriocins (Parada et al., 2007). Bacteriocins can colonize and persist in the human gut while having a beneficial effect on the host, including modulation of the composition of the gut microbiota, improvement of the host immune response, enhancement of the gut barrier function, and the modulation of immune system activities (Qiao et al., 2021). Collectively, these factors contribute to improved gut health, which subsequently enhances the productivity and quality of eggs produced by laying hens (Quarantelli et al., 2008; Mikulski et al., 2012; Mikulski et al., 2020; Shanmugam et al., 2024). Previous studies have indicated that PA can significantly improve both the production performance and egg quality of laying hens (Mikulski et al., 2012; Mikulski et al., 2020). However, the potential effects of PA on aged laying hens remain unclear. Therefore, the objective of this study is to elucidate the specific impacts of PA on the gut health of aged laying hens, ultimately aiming to improve laying performance and egg quality.

## Materials and methods

All experimental procedures were carried out in compliance with the guidelines established by the Animal Welfare Committee. Furthermore, the experimental protocol received approval from the Institutional Animal Care and Use Committee (IACUC) of Sichuan Agricultural University (No. 20181105).

### Probiotic preparation

*Pediococcus acidilactici* (CNCM I-4622 at 1 × 10^8^ CFU/g, Bactocell^®^; PA) was produced by Lallemand SAS (Blagnac, France).

### Experimental design

A total of 180 Lohmann pink laying hens at 80 weeks of age were housed individually in wire-layer cages. The hens were randomly allocated to five treatments with six replicates (six hens per replicate). PA was added in the basal diet (PA0) at concentrations of 50, 100, 150, and 200 mg/kg, respectively. A corn-soybean-meal basal diet in mash form was formulated to meet the nutrient requirements of hens as recommended by the National Research Council (1994) and the feeding standards for laying hens according to the Chinese Feeding Standard of Chicken (NY/T 33-2004) (Table 1). The study duration was 12 weeks (from 80 to 91 weeks of age) with free access to feed and water and a lighting schedule of 16 L:8 D.

**Table 1:** Ingredient composition and nutrient content of basal diets (g/kg, as-fed basis).

Item	Amount, %
Ingredients	
Corn	61.50
Soybean meal (CP43%)	23.60
Wheat bran	3.70
Soybean oil	0.62
Sodium chloride	0.40
Calcium carbonate	8.07
Calcium hydrophosphate	1.36
Mineral premix^1^	0.50
Vitamin premix^2^	0.03
DL-methionine	0.12
Choline chloride (50%)	0.10
Total	100.0
Analyzed nutrient content^3^	
Metabolizable energy, kcal/kg	2650.0
Crude protein, %	15.51
Calcium, %	3.51
Total phosphorus, %	0.59
Nonphytate phosphorus, %	0.32
Lysine, %	0.80
Methionine, %	0.37
Threonine, %	0.60
Tryptophan, %	0.18

1 Provided per kilogram of diet: copper (CuSO_4_·5H_2_O), 8 mg; manganese (MnSO_4_·H_2_O), 60 mg; iron (FeSO_4_·H_2_O), 60 mg; zinc (ZnSO_4_·H_2_O), 80 mg; iodine (KI), 0.35 mg; selenium (Na_2_SeO_3_), 0.3 mg.

2 Provided per kilogram of diet: vitamin A, 12,000 IU; vitamin D_3_, 3,000 IU; vitamin E, 30 IU; vitamin B_1_ (thiamine), 3 mg; vitamin B_2_ (riboflavin), 9.6 mg; vitamin B_6_, 6 mg; vitamin B_12_, 0.3 mg; biotin, 1.7 mg; pantothenic acid, 18 mg; folic acid, 1.5 mg; niacin, 60 mg.

3 Nutrient levels were calculated.

### Sample collection

At the end of week twelve, a total of 18 eggs from each group (3 eggs per replicate) were collected for egg quality assessment. The egg yolks were then carefully separated, with three yolks from each replicate mixed together and stored at −20°C.

At the end of the trial, 30 hens (one hen per replicate) were randomly selected. Blood samples were obtained via venipuncture from the wing vein using a sterile syringe, followed by centrifugation at 3,500 g for 10 min to isolate the serum, which was subsequently stored at −20°C until further analysis. Following this procedure, all selected hens were euthanized using carbon dioxide asphyxiation. The anterior segment of the jejunum and ileum was excised, and its contents were rinsed. The mucosal surface was scraped with a glass slide and placed into a cryopreservation tube, which was immediately frozen in liquid nitrogen and stored at −80°C. Approximately 2 cm sections from the middle of the ileum were excised and immersed in a 4% (v/v) formalin solution for histomorphometric measurement. The cecal chyme was initially stored in liquid nitrogen before being transferred to an ultra-low temperature refrigerator (−80°C) until analysis.

### Production performance

The number of dead hens, feed weight, egg count, egg weight, and the quantity of unqualified eggs (including dirty, malformed, broken, soft, and sand-shelled eggs) were recorded daily. Subsequently, the hen-day egg production rate and the hen-housed egg production rate, along with the average daily feed intake (ADFI), feed conversion ratio (FCR), average egg weight, and the qualified egg rate, were calculated on a weekly basis. The following formulas were employed:

Average daily feed intake (g/bird) = total feed consumption (g)/cumulative number of laying hens on the day of feeding; hen-housed egg production rate (%) = total number of eggs/cumulative number of laying hens at the beginning day of the trial × 100; hen-day egg production rate (%) = total number of eggs/cumulative number of laying hens per week × 100; feed conversion ratio = total feed consumption/total egg weight per week; average egg weight (g) = total egg weight/total number of eggs; egg mass (g/hen) = total cumulative egg weight/number of hens at the beginning day of the trial.

### Egg quality

The strength and thickness (blunt end, tip, and equator) of the eggshell were quantified using an eggshell strength tester and an eggshell thickness tester (Robotmation Co., Ltd., Tokyo, Japan). The color of the eggshell was assessed with a colorimeter (3NH-NR20XE, China). The internal quality of the egg was analyzed using an egg multi-tester (EMT-5200, Robotmation Co., Ltd., Tokyo, Japan), which provided measurements of the Haugh unit, albumen height, and yolk color. Additionally, the egg yolk was separated from the albumen using an egg separator, and the weight of the yolk was recorded. The egg yolk or eggshell index was calculated as 100 × [egg yolk or eggshell weight (g)/egg weight (g)]. The egg shape index was determined as the ratio of the longitudinal diameter to the transverse diameter.

### Fatty acids of egg yolk

The freeze-dried egg yolk sample was weighed and transferred to a homogenization tube. The tube was then filled with 0.8 mL of chloroform and 0.7 mL of a methanol-water solution. The sample was homogenized for 1 min, after which the supernatant was collected. An additional 2 mL of potassium hydroxide methanol solution was added to the supernatant to initiate the saponification reaction. After cooling the sample in an ice bath, 2 mL of boron trifluoride methanol was added, and the mixture was placed in a water bath at −80°C. Heating for 2 min completed the methyl esterification process. Once the mixture cooled to room temperature, 1 mL of *n*-hexane (0.05 g/L BHT *n*-hexane solution) and 2 mL of saturated NaCl solution were added, followed by the collection of the supernatant. The fatty acids in the egg yolk were analyzed using gas chromatography (GC-2010 Plus gas chromatograph coupled to a 2010 Plus single quadrupole mass spectrometer; Shimadzu Corp., Kyoto, Japan).

### Serum biochemical parameters

Alanine aminotransferase (ALT), aspartate aminotransferase (AST), urea nitrogen (UREA), total protein (TP), albumin (ALB), total cholesterol (TC), triglycerides (TG), high-density lipoprotein (HDL-C), and low-density lipoprotein (LDL-C) were measured using an automated biochemical analyzer (Hitachi 3100, China). All biochemical kits were from Nanjing Jiancheng Bioengineering Institute (Nanjing, China).

### ELISA assay

The concentrations of serum diamine oxidase (DAO), D-lactic acid (D-LA), and lipopolysaccharide (LPS) were determined using an enzyme-labeled instrument (Thermo Multiskan Ascent, United States) with an enzyme immunoassay.

Approximately 0.2 g of jejunal and ileal mucosa was collected, to which physiological saline was added at a ratio of 1:4 to create a 20% tissue homogenate. This mixture was then centrifuged at 3,000 r/min for 10 min, and the supernatant was collected. The activities of trypsin and chymotrypsin, as well as the concentration of secretory immunoglobulin A (SIgA), were quantified using an enzyme-linked immunosorbent assay kit obtained from Jiangsu Enzyme Immuno-Bio Co., Ltd. The preparation and handling of the reagents were performed in strict accordance with the provided instructions. The measurement of lipase and amylase was conducted using kits supplied by the Nanjing Jiancheng Bioengineering Institute (Nanjing, China).

### Intestinal morphology of ileum

The hematoxylin-eosin (H&E) staining and sealing were performed by Beijing Dacome Biotechnology Co., Ltd. Villus height (VH) and crypt depth (CD) were measured using a digital trinocular camera microscope (BA400 Digital, Japan). VH and CD were quantified in 10 villi from each slice, and the ratio of villus height to crypt depth (VH/CD) was subsequently calculated.

### RNA extraction and RT-qPCR

Total RNA was extracted from the jejunum mucosa using TRIzol reagent (TaKaRa, Dalian, China), and complementary DNA (cDNA) was synthesized with the PrimeScript RT reagent kit (Takara). Real-time PCR was conducted using SYBR Premix Ex Taq (Takara) on an Applied Biosystems 7900HT Real-Time PCR system (Applied Biosystems, Foster City, CA). The primer sequences for all genes are provided in Table 2.

**Table 2:** Primer sequences used to measure gene expression.

Genes	Orientation	Primer sequence (5′–3′)	Product size, bp	Accession number
*β-actin*	Forward	GCTACAGCTTCACCACCACA	133	NM_205518.1
	Reverse	TCTCCTGCTCGAAATCCAGT		
*Occludin*	Forward	GCTGAGATGGACAGCATCAA	104	NM_205128.1
	Reverse	CCTCTGCCACATCCTGGTAT		
*ZO-1*	Forward	GAGCGCAAGTTTGAAAGTCC	112	XM_015278981.2
	Reverse	AGGAGGCTGTGATGAGCTGT		
*MUC-2*	Forward	TGCCAGCCTTTTTATGCTCT	117	NM_001318434.1
	Reverse	AGTGGCCATGGTTTCTTGTC		
*IL-6*	Forward	CTCCTCGCCAATCTGAAGTC	90	NM_204628.1
	Reverse	CCCTCACGGTCTTCTCCATA		
*IL-8*	Forward	GATTGAACTCCGATGCCAGT	132	NM_205018.1
	Reverse	TCCACATTCTTGCAGTGAGG		
*IL-1*	Forward	GCATCAAGGGCTACAAGCTC	135	XM_015297469.1
	Reverse	CAGGCGGTAGAAGATGAAGC		
*IFN-γ*	Forward	CAGATGTAGCTGACGGTGGA	65	NM_205149.1
	Reverse	CATCGAAACAATCTGGCTCA		
*TNF-α*	Forward	GCCCTTCCTGTAACCAGATG	96	NM_204267.2
	Reverse	ACACGACAGCCAAGTCAACG		
*TLR-4*	Forward	GCCATTGCTGCCAACATCATCC	106	NM_001030693.2
	Reverse	ATGCCAGAGCGGCTACTCAGAA		
*MyD88*	Forward	CGTCGCATGGTGGTGGTTGTT	105	NM_001030962.5
	Reverse	TCGCTTCTGTTGGACACCTGGA		
*NF-κB*	Forward	TCAATGGCTACACAGGACCA	115	NM_001396395.1
	Reverse	CACTGTCACCTGGAAGCAGA		
*Claudin-1*	Forward	CATACTCCTGGGTCTGGTTGGT	77	NM_001013611.2
	Reverse	GACAGCCATCCGCATCTTCT		

*ZO-1, zonula occludens-1; MUC-2, mucin 2; IL, interleukin; IFN-γ, interferon-γ; TNF-α, tumor necrosis factor-α; TLR-4, Toll-like receptors-4; MyD88, myeloid differentiation primary response 88; NF-κB, nuclear factor kappa B.*

The RNA quality (intact ribosomal RNA 28 s/18 s) was evaluated by agarose gel electrophoresis, and RNA concentrations were subsequently quantified by spectrophotometer (NanoDrop 2000, Thermo Fisher Scientific). Complementary DNA (cDNA) was obtained by the reverse transcription process, and real-time polymerase chain reaction (PCR) was then performed in triplicate on an ABI 7500 Real-time PCR detection system (Applied Biosystems). This amplification program consisted of 95°C/15 min, followed by 40 cycles of 95°C/5 s and 60°C/30 s, and a final melting curve analysis. The *β-actin* was selected as the reference gene. The normalisation factor, which was subsequently used to normalise the relative amounts of RNA of interest, was obtained by calculating the geometric mean of the values of the selected reference genes.

### Analysis of SCFAs in the cecal contents by gas chromatography

The cecal contents must be accurately weighed and subsequently diluted with ultrapure water at a ratio of 1:8. The resulting mixture should be thoroughly shaken and mixed, after which the pH value should be measured using a portable pH meter (Testo 205, Testo AG, Schwarzwald, Germany).

A cecal content sample weighing 0.5 g should be placed into a centrifuge tube, followed by the addition of 1.2 mL of ultrapure water. The sample must then be vortexed for 3–5 min to ensure complete dissolution. After vortexing, the sample should be allowed to stand for 30 min, after which centrifugation at 10,000 g for 15 min should be conducted in the extraction solution. The resulting supernatant (1 mL) is to be combined with 0.2 mL of a 25% (w/v) metaphosphoric acid solution and 23.3 μL of a 210 mmol/L crotonic acid solution. The samples should be mixed thoroughly and incubated at 4°C for 30 min. Following incubation, centrifugation at 8,000 g for 10 min is to be performed. After centrifugation, 0.3 mL of the supernatant should be added to 0.9 mL of chromatographic methanol and mixed well, resulting in a 1:3 dilution. The sample should then be centrifuged at 8,000 rpm for 5 min. Finally, the supernatant should be filtered through a 0.22 μm filter into a 1.5 mL EP tube for subsequent analysis.

The precise weights of the following acids must be determined: 0.91 g of acetic acid, 0.37 g of propionic acid, 0.18 g of butyric acid, and isobutyric acid, as well as 0.20 g of valeric acid and isovaleric acid. These acids must then be dissolved in pure water and diluted to a final volume of 100 mL. The resulting concentrations were measured at 151.54, 50, and 20.03, respectively. Furthermore, the concentrations were calculated to be 20.03, 19.44, and 19.44 mmol/L.

The measurement was conducted using a gas chromatograph (CP-3800, Varian, United States). The chromatographic column employed was an HP-FFAP capillary column (30 m × 0.53 mm). The column was 53 mm in diameter and 1 μm in pore size. The temperature of the syringe or detector was 220°C, the injection volume was 1 μL, and the split ratio was 50:1. The carrier gas is high-purity N_2_, the column flow is 1 mL/min, the FID detector temperature is 250°C, the gas high-purity H_2_ flow is 40 mL/min, the gas-assisted zero-stage airflow is 400 mL/min, and the makeup gas is high-purity N_2_ flow rate 35 mL/min. The chromatographic column is HP-FFAP, and the column oven is programmed to rise in temperature as follows: starting at a temperature of 100°C, rising to 190°C at a rate of 20°C/min, holding for 0.5 min; the running time is 5 min. The calculation formula is as follows: *w* = *X* * 4 * 1.223 (mmol/L) * *V*_water_ * *M*_molecular weight_/*m*_0_.

### Analysis of intestinal microflora

The magnetic bead method for genomic DNA extraction from cecal contents was employed using a kit from Tiangen Biotech (Beijing) Co., Ltd., Beijing, China. After quantifying the extracted DNA, a polymerase chain reaction (PCR) analysis was conducted on the V4 variable region of the bacterial 16S rRNA using the following primers: forward primer, 5′-CCTAYGGGRBGCASCAG-3′; reverse primer, 5′-GGACTACNNGGGTATCTAAT-3′. The obtained PCR products were separated by electrophoresis in agarose gels (2%, w/v), and the target band’s product should be recovered using a universal DNA purification and recovery kit (Tiangen Biotech (Beijing) Co., Ltd., Beijing, China). The NEB Next^®^ Ultra^™^ II FS DNA PCR-free Library Prep Kit (New England Biolabs) was employed for library construction. The constructed library was quantified using Qubit and Q-PCR methods. Once qualified, the library underwent PE 250 on-machine testing on the NovaSeq 6000.

### Bioinformatic analysis

Delete the offline data based on the barcode sequence and PCR amplification primer sequence. The resulting spliced sequence is referred to as RawTags data. Utilize fastp software (Version 0.23.1) to conduct rigorous filtering on the spliced raw tags to obtain high-quality tags data, known as clean tags. The Uparse algorithm (Uparse v7.0.1001) was employed to cluster all effective tags from all samples. By default, the sequences were clustered into Operational Taxonomic Units (OTUs) with 97% similarity for subsequent species annotation. Finally, alpha diversity of the microbial community was calculated using QIIME (Version 1.9.1), while beta diversity was analyzed using R software (Version 4.0.3) to compare differences among the various treatment groups.

### Metabolomics analysis of cecal content

A volume of 300 μL of an 80:20 (v/v) methanol-water solution should be added to a sample of cecal content that has been previously homogenized and centrifuged at 14,000 g for 5 min. The resulting supernatant is then analyzed using liquid chromatography-mass spectrometry (LC-MS). The samples are subsequently transported to Novogene Bioinformatics Technology Co., Ltd. in Beijing, China, for further analysis.

### Statistical analysis

All data were analyzed using SAS 9.4 software. Differences between two groups were assessed using an independent sample *t*-test. For comparisons involving more than two groups, one-way ANOVA was employed, followed by Duncan’s test for multiple comparisons. Results are presented as the mean with the standard error of the mean (SEM). *p*-values <0.05 were considered significant, and 0.05 ≤ *p* < 0.10 was considered a tendency.

## Results

### Production performance

**Figure 1:**
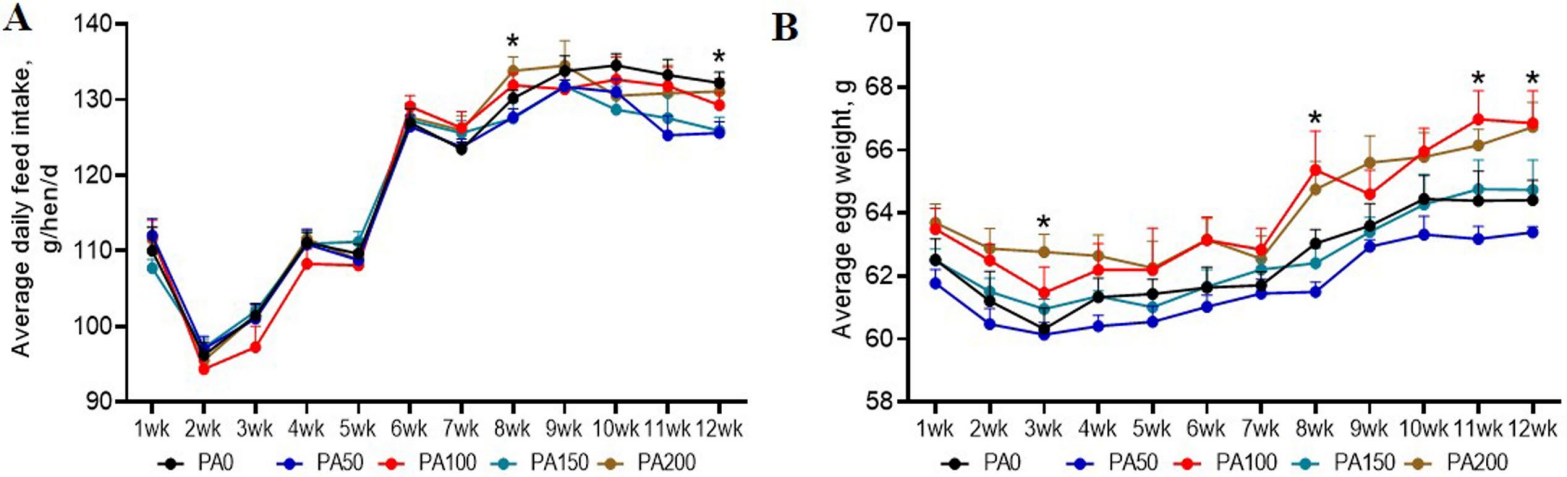
Effects of dietary supplementation of different levels of *Pediococcus acidilactici* on the production performance of aged laying hens during 1 to 12 weeks. **(A)** Average daily feed intake. **(B)** Average egg weight. Values are means and SEM, *n* = 6. Significant correlations are indicated by asterisks (^*^p < 0.05).

Throughout the entire study period, no significant differences were observed among the treatments with dead hens (1, 0, 2, 1, and 2 hens, respectively; data not shown). As indicated in Table 3, the addition of PA did not significantly affect the hen-day egg production rate, hen-housed egg production rate, ADFI, FCR, and egg mass during weeks 1 to 12 (*p* > 0.05). Notably, the ADFI in the PA50 group was significantly lower than that of the PA0 group at week 12 (*p* < 0.05, Figure 1A). Furthermore, the average egg weights in the PA100 and PA200 groups at weeks 3, 8, 11, and 12 were significantly higher than those in the PA0 group (*p* < 0.05, Figure 1B). The qualified egg rates in the PA50 and PA150 groups were significantly greater than those in the PA100 and PA200 groups (*p* = 0.022), but with no significant difference from the PA0 group. Additionally, the large egg rate (egg weight ≥70 g) in the PA100 and PA200 groups was significantly higher than that in the PA50 and PA150 groups (data not shown).

**Table 3:** Effects of dietary supplementation of different levels of PA on production performance of aged laying hens during 1 to 12 weeks.

Items	PA (mg/kg)					SEM	*p*-value		
	0	50	100	150	200		ANOVA	Linear	Quadratic
Hen-day egg production rate^1^, %	90.79	91.37	89.90	92.34	89.25	3.76	0.651	0.662	0.762
Hen-housed egg production rate^2^, %	89.39	91.37	86.57	90.64	87.10	2.43	0.562	0.494	0.772
Average daily feed intake, (g/hen/day)	120.02	118.44	118.86	118.47	120.32	2.67	0.654	0.963	0.376
Feed conversion ratio, g/g	2.12	2.10	2.07	2.05	2.11	0.07	0.450	0.394	0.276
Average egg weight, g	62.52^ab^	61.70^b^	63.96^a^	62.58^ab^	64.05^a^	1.33	0.022	0.046	0.129
Egg mass, g/hen	4,695	4,735	4,644	4,766	4,683	297	0.960	0.983	0.940
Qualified egg rate, %	86.45^ab^	89.66^a^	78.98^b^	90.49^a^	81.31^b^	6.39	0.015	0.339	0.623

Data were expressed as the mean and SEM. ^a–c^Value differences in the same row differ significantly (*p* < 0.05). (*n* = 6).

1 Hen-day egg production rate (%) = total number of eggs/cumulative number of laying hens per week × 100, This does not include any dead or culled birds.

2 Hen-housed egg production rate (%) = total number of eggs/cumulative number of laying hens at the beginning day of the trial × 100. This includes all dead or culled birds.

### Egg quality and fatty acid profile of egg yolk

As shown in Table 4, the egg yolk color of the PA150 group was significantly lower than that of the PA0 group, as well as the PA50, PA100, and PA200 groups (*p* = 0.011).

**Table 4:** Effects of dietary supplementation of different levels of PA on 12-week egg quality of aged laying hens.

Items		PA (mg/kg)					SEM	*p*-value		
		0	50	100	150	200		ANOVA	Linear	Quadratic
Eggshell color	*L* ^*^	82.24	82.58	83.59	83.31	83.43	1.62	0.544	0.134	0.257
	*a* ^*^	7.38	7.16	6.20	6.53	6.53	1.19	0.412	0.131	0.204
	*b* ^*^	14.23	14.10	12.51	13.36	13.28	1.65	0.389	0.223	0.288
Egg yolk color		13.25^a^	13.37^a^	13.41^a^	12.82^b^	13.21^a^	0.28	0.011	0.153	0.363
Eggshell strength, kg/cm^3^		3.71	3.48	3.64	3.43	3.37	0.56	0.813	0.298	0.587
Eggshell thickness, mm		0.378	0.368	0.336	0.365	0.362	0.012	0.168	0.200	0.252
Haugh unit		85.57	84.03	83.08	86.13	85.84	4.05	0.642	0.612	0.503
Egg shape index, %		1.314	1.332	1.343	1.323	1.316	0.013	0.508	0.916	0.250
Egg yolk ratio, %		29.20	29.19	26.45	26.76	27.55	3.95	0.627	0.347	0.391
Eggshell ratio, %		10.53	10.57	9.91	10.37	10.29	0.55	0.278	0.257	0.391

Data were expressed as the mean and SEM. ^a–c^Value differences in the same row differ significantly (*p* < 0.05). (*n* = 6).

Compared to the PA0 group, the content of C14:1 in the egg yolks of the PA150 group was significantly reduced (*p* = 0.013). Additionally, the content of C17:0 in the PA200 group was significantly decreased (*p* = 0.002), while the SFA in the PA200 group was significantly increased (*p* = 0.009, Table 5). The levels of C16:1 and the *n*−6/*n*−3 ratio in the egg yolk exhibited a linear relationship with the increasing PA concentration (*p* < 0.05). Conversely, C17:0, SFA, *n*−3 PUFA, and the UFA/SFA ratio demonstrated a quadratic relationship with the increasing PA concentration (*p* < 0.05).

**Table 5:** Effects of dietary supplementation of different levels of PA on 12-week fatty acids in egg yolks of aged laying hens.

Items	PA (mg/kg)					SEM	*p*-value		
	0	50	100	150	200		ANOVA	Linear	Quadratic
Myristic acid (C14:0)	0.305	0.291	0.318	0.298	0.314	0.008	0.120	0.361	0.620
Myristoleic acid (C14:1)	0.054^a^	0.051^ab^	0.058^a^	0.045^b^	0.059^a^	0.003	0.013	0.701	0.477
Pentadecanoic acid (C15:0)	0.044	0.049	0.047	0.047	0.048	0.003	0.740	0.597	0.733
Palmitic acid (C16:0)	24.89	24.78	25.23	24.91	25.26	0.269	0.631	0.303	0.586
Palmitoleic acid (C16:1)	2.45	2.44	2.71	2.55	2.81	0.118	0.139	0.032	0.101
Heptadecanoic acid (C17:0)	0.291^a^	0.319^a^	0.288^a^	0.280^a^	0.238^b^	0.012	0.002	0.002	0.001
Stearic acid (C18:0)	8.80	8.56	8.45	8.64	8.35	0.170	0.400	0.124	0.297
Oleic acid (C18:1c)	39.09	38.55	38.12	37.51	38.51	0.700	0.594	0.316	0.322
Linoleic acid (C18:2 *n*−6)	15.75	16.39	16.36	17.22	16.09	0.689	0.651	0.493	0.465
γ-Linolenic acid (C18:3 *n*−6)	0.156	0.166	0.134	0.173	0.155	0.015	0.416	0.930	0.953
α-Linolenic acid (C18:3 *n*−3)	0.505	0.542	0.567	0.526	0.493	0.033	0.538	0.698	0.225
Cis-11,14,17-Eicosatrienoic (C20:3 *n*−3)	0.011	0.012	0.012	0.013	0.011	0.001	0.374	0.903	0.203
Docosahexaenoic acid (C22:6 *n*−3)	1.12	1.09	1.15	1.07	1.02	0.035	0.109	0.068	0.059
SFA	34.34^b^	34.04^b^	34.37^b^	34.21^b^	35.41^a^	0.258	0.009	0.017	0.003
UFA	59.72	59.83	59.68	59.44	59.73	0.223	0.797	0.593	0.827
MUFA	41.85	41.30	41.13	40.35	41.64	0.654	0.548	0.510	0.368
PUFA	17.70	18.37	18.40	19.18	18.09	0.722	0.691	0.486	0.461
*n*−3 PUFA	1.64^ab^	1.65^ab^	1.74^a^	1.61^ab^	1.52^b^	0.046	0.048	0.097	0.020
*n*−6 PUFA	15.91	16.56	16.49	17.39	16.24	0.690	0.640	0.492	0.469
*n*−6/*n*−3	9.70	10.04	9.54	10.77	10.73	0.385	0.095	0.031	0.081
UFA/SFA	1.74	1.76	1.74	1.73	1.69	0.018	0.111	0.027	0.025

Data were expressed as the mean and SEM. ^a–c^Value differences in the same row differ significantly (*p* < 0.05). (*n* = 6).

### Serum indicators

Compared with the PA0 group, the serum TP, TG, and HDL-C contents of the PA100 group increased significantly (*p* < 0.05, Table 6). Conversely, the serum DAO levels in the PA50, PA100, PA150, and PA200 groups were significantly lower than those in the PA0 group (*p* < 0.05, Table 7). Additionally, the content of D-LA is the content of (*p* = 0.092). Notably, both TG and DAO serum levels increased with rising PA concentrations, demonstrating a quadratic curve change (*p* < 0.05).

**Table 6:** Effects of dietary supplementation of different levels of PA on serum biochemical indicators of aged laying hens.

Items	PA (mg/kg)					SEM	*p*-value		
	0	50	100	150	200		ANOVA	Linear	Quadratic
TP, g/L	51.80^b^	53.05^b^	58.99^a^	52.61^b^	54.00^b^	1.71	0.048	0.519	0.198
ALB, g/L	18.63	21.15	21.49	20.96	20.38	0.93	0.233	0.279	0.073
ALT, U/L	4.23	4.29	5.49	4.86	4.26	0.38	0.113	0.637	0.104
AST, U/L	180.60	161.46	165.17	166.28	155.54	9.23	0.421	0.123	0.285
TC, mmol/L	2.94	2.61	3.54	2.96	3.03	0.41	0.628	0.679	0.826
TG, mmol/L	11.90^b^	14.10^b^	19.70^a^	16.84^ab^	16.87^ab^	1.74	0.040	0.038	0.020
HDL-C, mmol/L	1.57^b^	1.74^b^	2.47^a^	1.81^b^	1.90^b^	0.17	0.014	0.271	0.066
LDL-C, mmol/L	1.86	1.91	2.33	1.97	2.07	0.19	0.426	0.444	0.477
UREA, mmol/L	0.23	0.21	0.26	0.20	0.21	0.02	0.270	0.408	0.640

Data were expressed as the mean and SEM. ^a–c^Value differences in the same row differ significantly (*p* < 0.05). (*n* = 6).

**Table 7:** Effects of dietary supplementation of different levels of PA on serum intestinal permeability indicators of aged laying hens.

Items	PA (mg/kg)					SEM	*p*-value		
	0	50	100	150	200		ANOVA	Linear	Quadratic
LPS, ng/L	92.63	90.76	85.28	88.03	81.79	6.50	0.777	0.222	0.481
DAO, pg/mL	151.10^a^	106.15^b^	120.20^b^	103.68^b^	109.55^b^	8.78	0.005	0.010	0.006
D-LA, ng/L	347.13	295.69	300.29	334.19	290.81	16.86	0.092	0.205	0.364

Data were expressed as the mean and SEM. ^a–c^Value differences in the same row differ significantly (*p* < 0.05). (*n* = 6).

### Intestinal morphology and digestive enzyme activity

**Figure 2:**
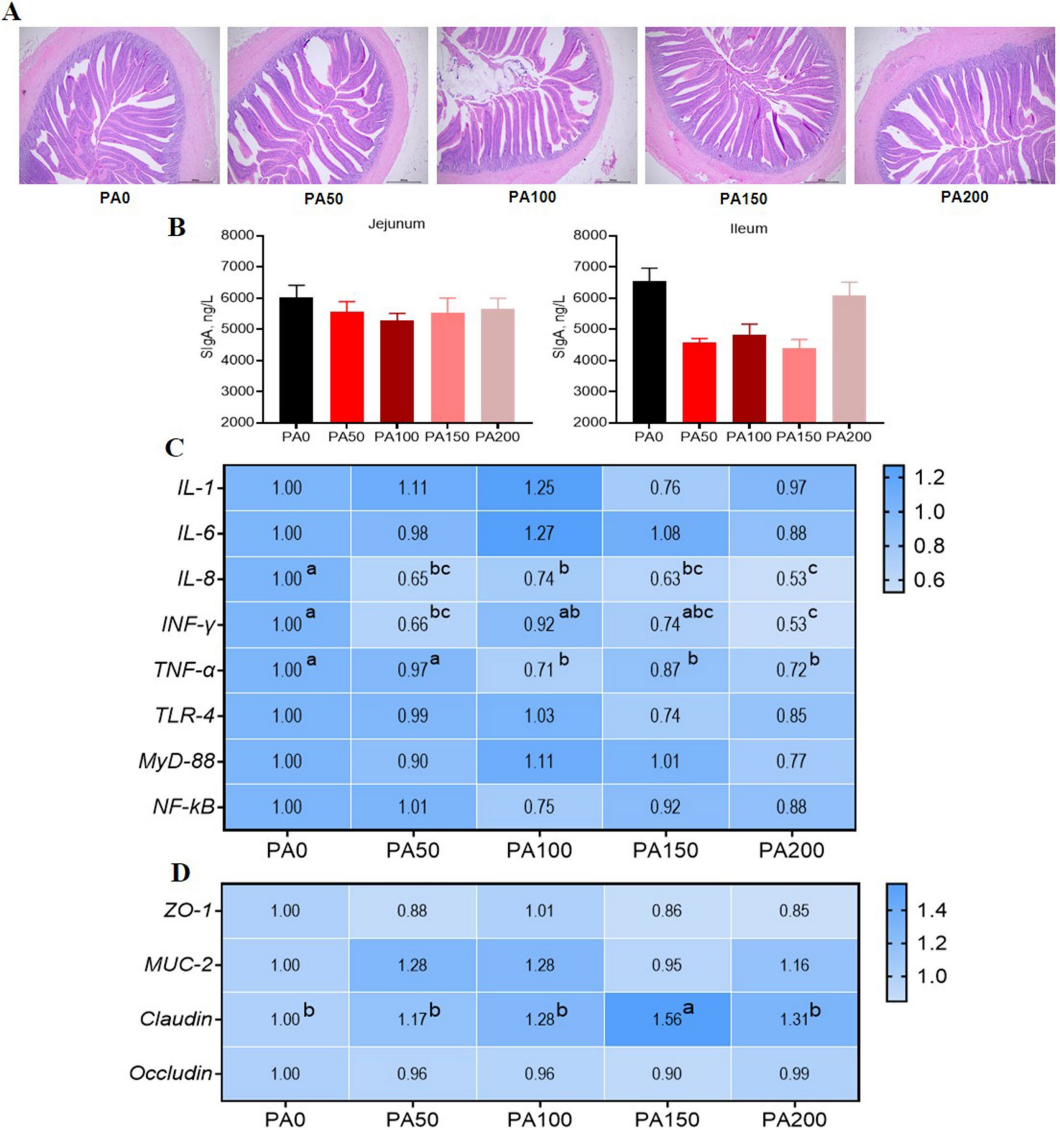
Effects of dietary supplementation of different levels of *Pediococcus acidilactici* on intestinal morphology and gene expression of aged laying hens. **(A)** Ileum H&E staining. **(B)** Intestinal mucosa secretory immunoglobulin A. **(C)** Jejunal mucosal inflammatory factor gene expression. **(D)** Jejunal mucosal barrier function gene expression. The larger the value, the bluer the color. Values are means and SEM, *n* = 6. Different letters indicate significant differences at *p* < 0.05. Significant correlations are indicated by asterisks (^*^p < 0.05).

The ileal VH in the PA50, PA100, PA150, and PA200 groups was significantly higher than that in the PA0 group (*p* = 0.001). The CD of the PA100 and PA150 groups was significantly lower than that in the PA0 group (*p* = 0.005). Ileal VH exhibited a linear increase with rising PA concentration (*p* < 0.001), while CD demonstrated a quadratic change in response to increasing PA concentration (*p* = 0.001, Table 8). As illustrated in Figure 2B, the SIgA content in the jejunal and ileal mucosa of the PA50, PA100, PA150, and PA200 groups was lower than that in the PA0 group (*p* > 0.05). Compared with the PA0 group, lipase activity in the jejunum of the PA200 group was significantly increased (*p* = 0.036), and lipase activity in the ileum of the PA150 group was significantly increased (*p* = 0.012). Conversely, chymotrypsin activity in the ileum of the PA50 group was significantly decreased (*p* = 0.004). Both chymotrypsin activity in the ileal mucosa and lipase activity exhibited a quadratic change with increasing PA concentration (*p* < 0.05, Table 9).

**Table 8:** Effects of dietary supplementation of different levels of PA on ileum morphology of aged laying hens.

Items	PA (mg/kg)					SEM	*p*-value		
	0	50	100	150	200		ANOVA	Linear	Quadratic
VH/μm	757.01^b^	872.09^a^	844.11^a^	917.23^a^	928.70^a^	27.11	0.001	<0.001	0.001
CD/μm	133.79^a^	123.34^ab^	111.59^b^	116.81^b^	133.20^a^	4.46	0.005	0.673	0.001
VH/CD	6.32	7.73	7.15	8.18	7.65	0.48	0.105	0.057	0.081

Data were expressed as the mean and SEM. ^a–c^Value differences in the same row differ significantly (*p* < 0.05). (*n* = 6).

**Table 9:** Effects of dietary supplementation of different levels of PA on the activity of intestinal digestive enzymes in aged laying hens.

Items	PA (mg/kg)					SEM	*p*-value		
	0	50	100	150	200		ANOVA	Linear	Quadratic
Jejunum									
Trypsin activity, U/L	3901.4	3206.6	3658.9	3531.2	4034.2	366.4	0.547	0.614	0.367
Chymotrypsin activity, U/L	1112.8	1087.5	924.8	1167.1	1057.4	65.2	0.136	0.890	0.668
Lipase activity, U/gprot	2.66^a^	2.12^ab^	2.11^ab^	2.52^a^	1.79^b^	0.20	0.036	0.061	0.179
α-Amylase activity, U/mgprot	0.25	0.26	0.19	0.27	0.24	0.03	0.483	0.930	0.856
Ileum									
Trypsin activity, U/L	3106.2	3145.2	2857.3	3192.1	2836.7	220.5	0.681	0.477	0.764
Chymotrypsin activity, U/L	1038.3^ab^	838.5^c^	974.8^bc^	1142.3^ab^	1202.9^a^	64.10	0.004	0.009	0.004
Lipase activity, U/gprot	1.51^b^	2.00^ab^	2.00^ab^	2.47^a^	1.75^b^	0.18	0.012	0.162	0.013
α-Amylase activity, U/mgprot	0.182	0.153	0.175	0.186	0.195	0.01	0.295	0.185	0.202

Data were expressed as the mean and SEM. ^a–c^Value differences in the same row differ significantly (*p* < 0.05). (*n* = 6).

### Intestinal inflammatory factors and barrier function related mRNA expression

As illustrated in Figure 2C, the expression of *IL-8* mRNA in the jejunal mucosa was significantly reduced in the PA50, PA100, PA150, and PA200 groups when compared to the PA0 group (*p* < 0.05). Additionally, the expression of *INF-γ* mRNA was significantly diminished in the PA50 and PA200 groups (*p* < 0.05). Furthermore, the expression of *TNF-α* mRNA was significantly lower in the PA100, PA150, and PA200 groups (*p* < 0.05). Notably, the expression of *Claudin* mRNA in the PA150 group was significantly higher than that observed in the PA0 group (*p* < 0.05, Figure 2D).

### Analysis of SCFAs in the cecal contents by gas chromatography

**Figure 3:**
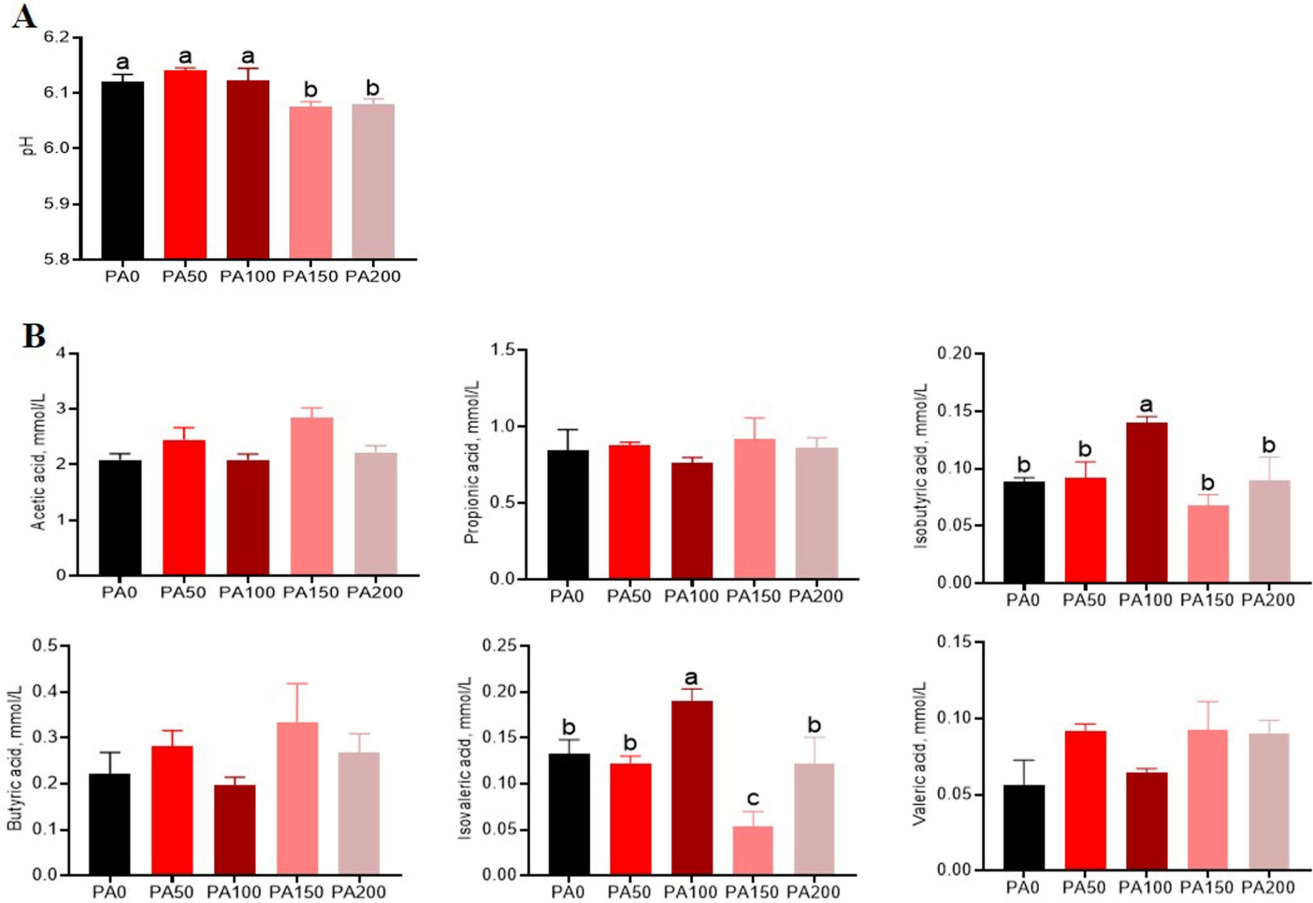
Effects of different concentrations of *Pediococcus acidilactici* on pH and volatile fatty acids in the cecal contents of aged laying hens. **(A)** pH value. **(B)** Short-chain fatty acids. Values are means and SEM, *n* = 6. Different letters indicate significant differences at *p* < 0.05.

The pH value of cecal contents in the PA150 and PA200 groups was significantly lower than that observed in the PA0 group, as well as in the PA50 and PA100 groups (*p* < 0.05, Figure 3A). Additionally, when compared to the PA0 group, the levels of isobutyric acid and isovaleric acid were significantly elevated in the PA100 group (*p* < 0.05, Figure 3B).

### Microbial analysis of cecal contents

**Figure 4:**
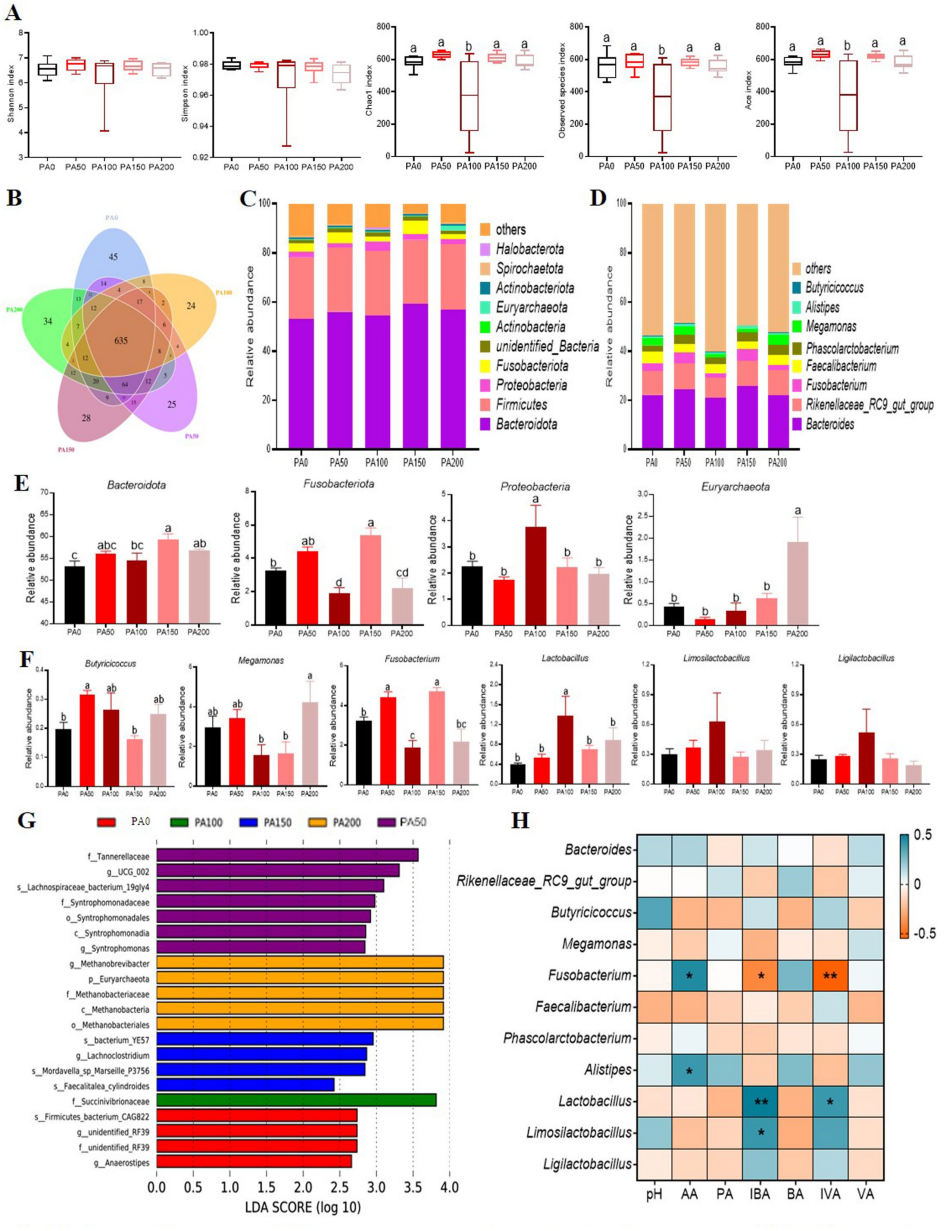
Effects of different concentrations of *Pediococcus acidilactici* on the microbial structure of the cecal contents of aged laying hens. **(A)** Alpha diversity index. **(B)** Venn diagram of operational taxonomic units (OTUS). **(C,D)** The cecal bacterial community compositions at phylum and genus levels. **(E)** Resultant composition of microorganisms in cecal contents (phylum level). **(F)** Resultant composition of microorganisms in cecal contents (genus level). **(G)** Microbial LefSe analysis of the cecum. **(H)** Pearson correlation coefficient analysis of intestinal microorganisms with short-chain fatty acids. Blue and yellow cells represent positive and negative correlations, respectively. AA, acetic acid; PA, propionic acid; IBA, isobutyric acid; BA, butyric acid; IVA, isovaleric acid; VA, isovaleric acid. Values are means + SEM, *n* = 6. Different letters indicate significant differences at *p* < 0.05. Significant correlations are indicated by asterisks (^*^p < 0.05 and ^**^p < 0.01).

The Chao1, observed species, and Ace indices of the PA100 group were significantly lower than those of the PA0 group and the PA50, PA150, and PA200 groups (*p* < 0.05, Figure 4A). Among a total of 1077 OTUs, 58.96% (635 core OTUs) were shared among the five groups (Figure 4B). In contrast, the relative abundance of *Bacteroidota* in the PA150 and PA200 groups was significantly higher than that in the PA0 group (*p* < 0.05, Figures 4C,E). Additionally, the relative abundance of *Proteobacteria* in the PA100 group was significantly elevated compared to the PA0 group as well as the PA50, PA150, and PA200 groups (*p* < 0.05). The relative abundance of *Fusobacteriota* was significantly lower in the PA100 and PA150 groups than in the PA0 group (*p* < 0.05, Figures 4D,F). Furthermore, the relative abundance of *Euryarchaeota* was significantly higher in the PA200 group compared to the PA0, PA50, PA100, and PA150 groups (*p* < 0.05). The relative abundance of *Lactobacillus* in the PA100 group was significantly greater than that in the PA0, PA50, and PA150 groups (*p* < 0.05). Interestingly, the relative abundances of *Lactobacillus* (*p* < 0.05), *Ligilactobacillus* (*p* > 0.05), and *Limosilactobacillus* (*p* > 0.05) were observed in the PA group. To further investigate the differences in intestinal flora structure among the various treatment groups, linear discriminant analysis effect size (LefSe) analysis was performed on the cecal contents of the hens, with results displayed in Figure 4G. The PA50 group exhibited the most significant differences in microbial composition, particularly with f_*Tannerellaceae* being significantly enriched. In the PA100 group, the only microorganism that showed significant differences was f_*Succinivibrionaceae*.

The Pearson correlation coefficient analysis of intestinal microorganisms and short-chain fatty acids reveals that acetic acid is positively correlated with *Fusobacterium* and Alistipes (*p* < 0.05, Figure 4H). Isobutyric acid shows a positive correlation with *Lactobacillus* (*p* < 0.01) and *Limosilactobacillus* (*p* < 0.05), while exhibiting a negative correlation with *Fusobacterium* (*p* < 0.05). Additionally, isovaleric acid is positively correlated with *Lactobacillus* (*p* < 0.05) and negatively correlated with *Fusobacterium* (*p* < 0.01).

### Metabolomics analysis of cecal contents

**Figure 5:**
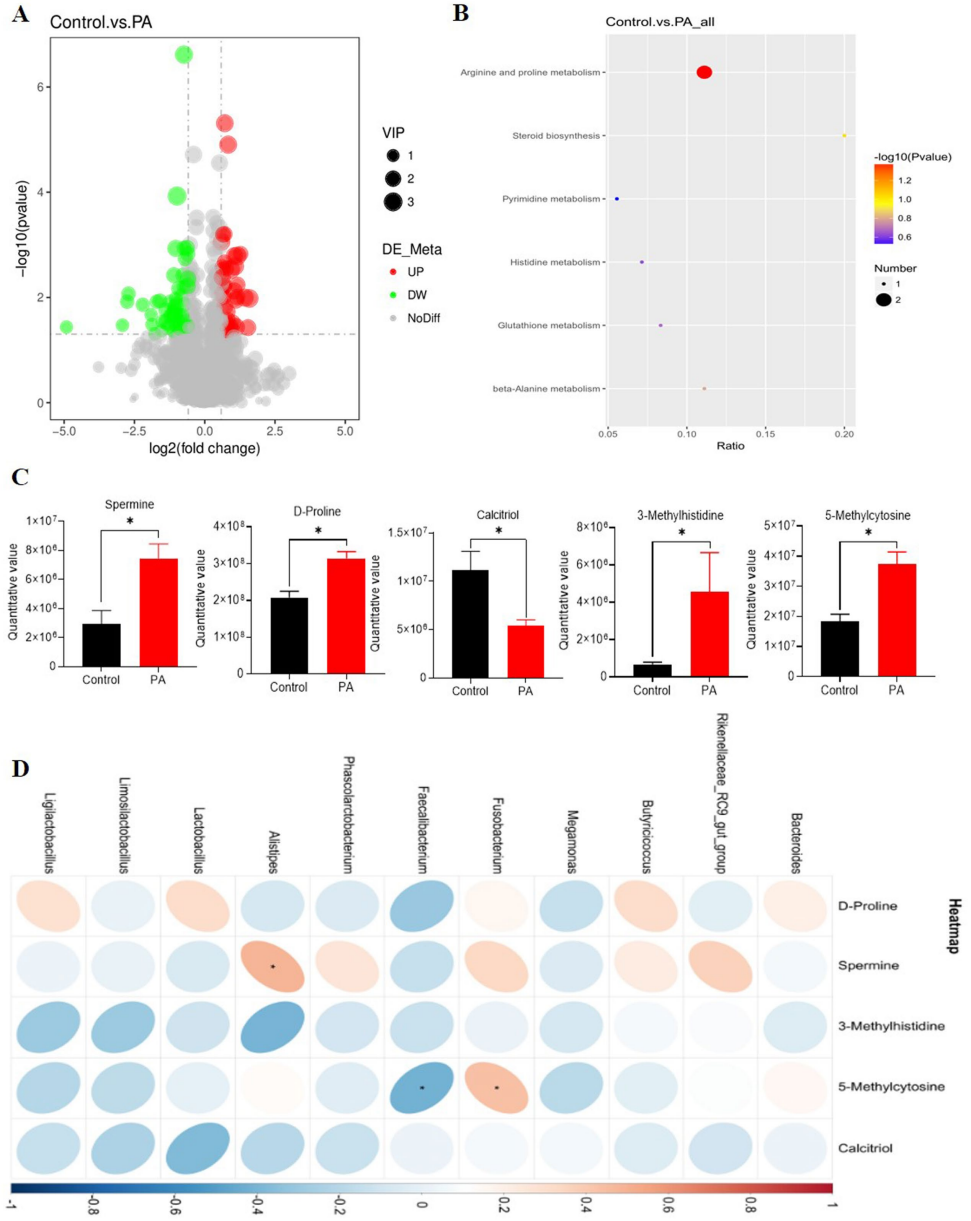
Effects of *Pediococcus acidilactici* supplementation on metabolomic profiles of cecal digesta of aged laying hens. **(A)** Differential metabolite volcanogram. The significantly up-regulated metabolites are represented by red dots, while down-regulated metabolites are represented by green dots. **(B)** Cecal chyme differential metabolites enriched by the Kyoto Encyclopedia of Genes and Genomes (KEGG). The color of the spot indicates the *p*-value. The redder, the more significant the enrichment. The size of the spot represented the number of different metabolites enriched. **(C)** Differential metabolites. ^*^The value difference between the control group and PA group is significant (*p* < 0.05). **(D)** Correlation between differential metabolites and bacterial genera in the cecal digesta of laying hens. Red and blue cells represent positive and negative correlations, respectively. Significant correlations are indicated by asterisks (^*^p < 0.05). Control: no PA group; PA: combination of PA50, PA100, PA150, and PA200 groups.

The results of the partial least squares discrimination analysis indicate that each group of models is well-established. The metabolite volcano plot comparing the PA and control groups revealed a total of 91 metabolites with significant differences. Among these, 39 metabolites were found to be upregulated, while 52 were downregulated (Figure 5A). All metabolites identified in the cecal contents were matched to the KEGG database to obtain information regarding the pathways in which these metabolites participate. The arginine and proline metabolic pathways emerged as the most enriched pathways for the differential metabolites (Figure 5B). Within the arginine and proline metabolism pathways, we observed that PA significantly increased the levels of spermine and D-proline (*p* < 0.05). Additionally, it significantly elevated the contents of 3-methylhistidine and 5-methylcytosine (*p* < 0.05), while significantly decreasing the level of calcitriol (*p* < 0.05, Figure 5C). In the correlation analysis between metabolomics and 16S, among these differential metabolites, spermine exhibited a significant positive correlation with *Alistipes* (*p* < 0.05). Conversely, *Faecalibacterium* and *Fusobacterium* demonstrated a significant negative correlation with 5-methylcytosine (*p* < 0.05, Figure 5D).

## Discussion

The dietary probiotics have been shown to improve both egg production and egg quality by reducing pathogenic bacterial loads and preventing damage to the gut structure (Zhang et al., 2012; Forte et al., 2016; Upadhaya et al., 2016). The results of this study suggest that incorporating PA (CNCM I-4622) into the diet of laying hens can improve egg production rates and FCR, particularly at a dosage of 150 mg/kg. Additionally, our study found that while the inclusion of 100 mg/kg and 200 mg/kg of PA increased average egg weight, it also led to a decrease in the egg production rate. A recent study corroborated that the addition of 100 mg/kg of PA significantly improved the egg production rate of laying hens (Mikulski et al., 2020). In prior research, it was observed that a diet supplemented with 100 mg/kg of PA resulted in notable increases in egg mass and average egg weight, alongside a reduction in the feed conversion ratio (Mikulski et al., 2012). Previous reports have indicated that probiotics can improve nutrient absorption by enhancing the intestinal environment of animals, thereby contributing to increased egg weight (Nahashon et al., 1994). The observed increase in egg weight was primarily attributed to a higher proportion of eggshell and greater eggshell thickness, which subsequently reduced the rate of damaged eggs (Mikulski et al., 2012; Mikulski et al., 2020). Furthermore, Mikulski et al. (2020) noted that improvements in egg weight and shell quality may be linked to enhanced calcium absorption and retention associated with probiotic supplementation.

In our study, 150 mg/kg PA significantly decreased the color of egg yolk. Previous research has indicated that feed composition and conversion rates can influence egg yolk color, with carotenoid content in eggs also playing a crucial role (Zhao et al., 2019). Consequently, the impact of probiotics on egg yolk color necessitates further investigation. Our findings demonstrate that PA alters the levels of myristoleic acid, heptadecanoic acid, SFA, and *n*−3 PUFA in egg yolk. An earlier study revealed that the addition of the PA strain MA18/5 M (consistent with CNCM I-4622 composition) at 100 mg/kg led to a significant decrease in palmitoleic acid content in egg yolk, accompanied by notable increases in margaric, margaroleic, and linoleic acids, as well as a substantial rise in overall PUFA and *n*−6 PUFA content (Mikulski et al., 2012). Saleh et al. (2016) reported that the incorporation of lactic acid bacteria in feed significantly reduced palmitoleic acid levels in egg yolks. Conversely, this approach markedly elevated the concentrations of oleic acid, linoleic acid, palmitoleic acid, gamma-linolenic acid, arachidonic acid, and nervonic acid, resulting in an overall increase in polyunsaturated fatty acids and a decrease in saturated fatty acids. Research suggests that the mechanism by which probiotics elevate unsaturated fatty acids in the intestine may be linked to amino acids and the activity of lactic acid bacteria (Saleh et al., 2013). Similar studies by Haddadin et al. (1996) and Ramasamy et al. (2008) indicated that *Lactobacillus* had a minimal effect on the fatty acid composition of egg yolks, which may be attributed to the specific type of probiotic used and the dosage administered (Abdelqader et al., 2013a; Mikulski et al., 2020).

Serum biochemical indicators serve as reflections of alterations in tissue cell permeability and metabolic function within the body, making them sensitive markers of animal health status (Jia et al., 2019). This study found that the addition of 100 mg/kg significantly elevated serum TP, TG, and HDL-C levels. These findings align with previous research. Specifically, studies have demonstrated that probiotics enhance serum/plasma immunoglobulin levels and induce changes in immune cell populations and their phagocytic capabilities (Peralta-Sánchez et al., 2019). HDL-C serves as an indicator of excess cholesterol being excreted into the intestines (Wang W. W. et al., 2020). Consequently, this study indicates that incorporating 100 mg/kg of PA into the diet not only increased serum TG levels but also facilitated the excretion of surplus cholesterol into the intestine, thereby regulating blood lipid metabolism. Additionally, endotoxins, commonly referred to as lipopolysaccharides, are components of the cell wall of Gram-negative bacteria (Wang et al., 2021). D-LA, a byproduct of bacterial metabolism, has been proposed as a potentially valuable marker for assessing the extent of intestinal damage and gut barrier dysfunction (Murray et al., 1993). DAO, an enzyme present in elevated concentrations in the intestinal mucosa of humans and other mammals, serves as a marker for mucosal maturation and integrity (Lei et al., 2013). Our findings indicate that the addition of PA significantly reduced serum concentrations of DAO, consistent with the results reported by Lei et al. (2013). This suggests that intestinal permeability is largely dependent on the integrity of the intestinal barrier. Intracellular DAO, which acts as a biomarker for intestinal permeability, can be released into the circulatory system in substantial quantities. When there is a breach in the integrity of the intestinal barrier, the levels of DAO in the blood increase (Wu et al., 2022). Damage to the intestinal epithelial barrier is evidenced by the presence of abnormally tight junctions and increased epithelial permeability, leading to the influx of greater quantities of DAO, D-LA, and LPS into the peripheral circulation (Deng et al., 2023).

The morphology of the small intestine and the activity of digestive enzymes are critical indicators for assessing the digestive and absorptive functions in poultry. A reduction in VH and CD may lead to impaired nutrient absorption, increased secretion within the gastrointestinal tract, and reduced performance (Xu et al., 2003). In contrast, an increase in VH and the V/C is positively correlated with enhanced epithelial cell turnover. Previous studies have shown that probiotic supplementation can stimulate the proliferation of intestinal epithelial cells in chickens (Mappley et al., 2011). Yang et al. (2020) demonstrated that the dietary inclusion of *Clostridium butyricum* can significantly improve the VH and V/C ratios in the duodenum, jejunum, and ileum of laying hens. The results of our study indicated that the addition of PA significantly increased the height of ileal VH in laying hens while reducing the CD. Furthermore, PA was found to significantly increase the V/C. The observed improvement in the intestinal mucosal structure in the PA treatment group may lead to improved nutrient absorption and increased disease resistance, a finding supported by measurements of digestive enzyme activity. Previous research has demonstrated that probiotics can improve dietary digestibility by producing hydrolytic enzymes that facilitate nutrient absorption, including phytase, lipase, amylase, and protease (Wang and Gu, 2010; Flint et al., 2012). The existing literature on the effects of probiotics on intestinal digestive enzymes is extensive. For instance, a study found that the administration of *Clostridium butyricum* to the diet of broilers infected with *Escherichia coli* K88 resulted in enhanced activities of amylase, protease, and lipase (Zhang et al., 2016). This finding was also corroborated by Jiang et al. (2009). Our study’s results indicate that the addition of 150 mg/kg and 200 mg/kg PA significantly increased lipase activity and chymotrypsin activity in the ileal mucosa, respectively. Ogawa et al. (2015) demonstrated that *Lactobacillus aerogenes* SBT2055 significantly reduced lipase activity, increased the size of lipid emulsion droplets, and inhibited lipid absorption. This finding contrasts with the results of Gong et al. (2018), who showed that the addition of probiotics to basal diets can markedly enhance amylase and lipase activities. In their studies, Rodjan et al. (2018) and Wang et al. (2021) found that the inclusion of *Bacillus subtilis* did not significantly affect the lipase activity in broiler chickens. This discrepancy may be attributed to the varying efficacy of different probiotic strains in enzyme production and/or the stimulation of endogenous enzyme production in broilers (Wang and Gu, 2010). Additionally, differences in pH levels within the gastrointestinal tract may also contribute to these outcomes. It is well established that the pH of the gastrointestinal tract significantly influences nutrient absorption and the gut microbiome in birds (Liu et al., 2021). The activity of various digestive enzymes is contingent upon maintaining a specific pH range, which is essential for the enzymes to perform their digestive and absorptive functions (Wang et al., 2021).

Probiotics have been shown to protect the intestinal epithelium by preventing the entry of pathogens into the gut (Vicente et al., 2008), inducing the expression of intestinal protective factors (Lutgendorff et al., 2009), strengthening tight junctions (Mennigen et al., 2009), and reinforcing the cytoskeleton of epithelial cells (Sherman et al., 2005). SIgA is the primary antibody found in intestinal secretions and is the most prevalent immunoglobulin in the mucosa. Furthermore, SIgA plays a crucial role in the formation of SIgA-coated bacterial complexes, which are essential for maintaining intestinal immune homeostasis (Macpherson et al., 2008). In our study, the addition of PA reduced the concentration of SIgA in the jejunum and ileum, thereby maintaining intestinal immune balance. Additionally, we found that PA significantly decreased the expression of pro-inflammatory factors *IL-8*, *INF-γ*, and *TNF-α* in the jejunal mucosa. Furthermore, 150 mg/kg also significantly elevated the expression of *Claudin*. Several studies have demonstrated that the consumption of probiotics can suppress the expression of pro-inflammatory cytokines while enhancing the expression of anti-inflammatory cytokines (Hu et al., 2021; Wang et al., 2021). The production of inflammatory cytokines can bolster the immune response, providing protection against pathogen invasion. However, an excessive inflammatory response may lead to tissue damage. Collectively, these findings suggest that probiotics may stimulate the immune response to protect against pathogen intrusion and thus sustain intestinal equilibrium by maintaining a delicate balance between pro-inflammatory and anti-inflammatory reactions.

SCFAs are a class of saturated fatty acids characterized by having fewer than six carbon atoms. In addition to serving as the primary energy source for colonocytes, substantial evidence suggests that SCFAs play a pivotal role in maintaining health and modulating immune and inflammatory responses (van der Hee and Wells, 2021). A study conducted by Xu et al. (2021) demonstrated that the dietary inclusion of probiotics can elevate the levels of various SCFAs in cecal contents. Consistent with this research, our study indicated that the addition of PA resulted in a significant reduction in cecal chyme pH; this observation may be attributed to the increase in SCFA concentrations and alterations in gut microbial structure. The incorporation of PA into the diet of laying hens has been shown to enhance the concentrations of acetic acid, propionic acid, butyric acid, valeric acid, and isobutyric acid in the cecal chyme. Furthermore, research indicates that the observed increase in immunoglobulins and alterations in cytokine levels may be associated with enhanced SCFA production (Parada Venegas et al., 2019; Luu et al., 2020).

The significance of the commensal gut microbiota for the normal functioning of the gut’s immune system is paramount (Neish, 2009; Adeoye et al., 2016). Numerous studies have demonstrated that probiotics can positively influence the composition of the intestinal microbiota (Kristensen et al., 2016). Specifically, probiotic supplementation has been shown to significantly alter the overall structure of the fecal bacterial community, particularly in terms of β-diversity (Kristensen et al., 2016). It is well established that the intestinal microbiota has several physiological effects on the host, including the metabolization of dietary nutrients, the production of SCFAs from indigestible carbohydrates, the synthesis of amino acids and vitamins, and the regulation of metabolism (Kamada et al., 2012). The addition of PA resulted in the Simpson index, Chao1 index, observed species index, and ACE index in cecal chyme. At the phylum level, varying concentrations of PA altered the relative abundances of *Bacteroidota*, *Fusobacteriota*, *Proteobacteria*, and *Euryarchaeota* in the cecal digesta of laying hens. Similarly, at the genus level, different concentrations of PA affected the relative abundances of *Butyricicoccus*, *Megamonas*, *Fusobacterium*, and *Lactobacillus*. Notably, PA increased the relative abundances of *Fusobacteriota* and *Euryarchaeota* at the phylum level while significantly enhancing the relative abundances of *Fusobacterium*, *Phascolarctobacterium*, and *Lactobacillus* at the genus level. Research indicates that *Fusobacteriota* and *Euryarchaeota* metabolize carbohydrates into SCFAs, which confer several benefits to the host, including the provision of energy to gastrointestinal cells (Wang et al., 2022; Zhu et al., 2022a). Furthermore, *Bacteroidetes* play a crucial role in regulating lipid and bile acid metabolism, as well as maintaining energy balance in the host (Zhu et al., 2022c). Furthermore, research has demonstrated that incorporating probiotics into the diet can significantly enhance the concentration of *lactic acid* bacteria (Mountzouris et al., 2010) and improve the activity of the cecal microflora (Mountzouris et al., 2007). This indicates that probiotics selectively promote specific taxa (Schaub et al., 2022). In our study, an increase in PA concentration was accompanied by a corresponding rise in the relative abundance of *Lactobacillus* in cecal digesta, which may also contribute to the significant decrease in pH observed. These results align with findings from Lei et al. (2015) and Yang et al. (2012). Additionally, several studies have shown that *lactic acid* bacteria can inhibit the colonization of *Campylobacter* in the digestive tract of chickens (Kobierecka et al., 2017). Consequently, PA has the potential to significantly reduce the colonization of harmful bacteria within the intestinal tract, increase the concentration of beneficial bacteria, and optimize the intestinal microenvironment of laying hens, thereby enhancing production performance.

Recent investigations have demonstrated a close relationship between the host’s gut microbiota and metabolic pathways (Wang D. et al., 2020; Zhang J. et al., 2021; Zhang X. et al., 2021). Metabolomics, a novel analytical approach, enables the detection of changes in small endogenous metabolites that may be influenced by external factors or internal disruptions. This technique can be utilized to diagnose and predict the underlying mechanisms of these alterations (Vang et al., 2022; Von-Hafe et al., 2022). Analysis of the metabolomic data reveals that the differential metabolites following the addition of PA are primarily enriched in the arginine and proline metabolism pathways. These pathways have been proposed to be closely linked to inflammation (Wijnands et al., 2015). The study indicated that several metabolites, including spermine, D-proline, 3-methylhistidine, and 5-methylcytosine, were significantly upregulated upon the addition of PA. Notably, amino acid metabolism is a critical regulator of inflammation and atherosclerosis, with arginine and proline metabolism identified as the key pathways relevant to SII and hs-CRP (Zhu et al., 2022b). Furthermore, arginine is ultimately converted into putrescine, proline, and glutamine through the action of specific enzymes. Putrescine can be transformed into spermidine and spermine, while glutamine can enter the TCA cycle and be oxidized to provide energy, generating CO_2_ (Zhang et al., 2022). Therefore, it is hypothesized that PA may alleviate intestinal inflammation in chickens by modulating the metabolic pathways of arginine and proline.

## Conclusion

In conclusion, this study demonstrates that the inclusion of PA in the diet of the aged laying hen improves egg production by improving intestinal morphology, digestive enzyme activity, and intestinal permeability, as well as increasing SCFAs and the intestinal microbial structure in laying hens. These improvements may be linked to the metabolic pathways of arginine and proline. Ultimately, these findings contribute to a deeper understanding of the mechanisms through which dietary PA regulates both production performance and intestinal health in laying hens.

## Data Availability

The datasets generated for this study can be found in The National Center for Biotechnology Information (NCBI), (Accession No. PRJNA1211985).
